# Ferroptosis and its role in skeletal muscle diseases

**DOI:** 10.3389/fmolb.2022.1051866

**Published:** 2022-11-03

**Authors:** Ying Wang, Zepeng Zhang, Weikai Jiao, Yanyan Wang, Xiuge Wang, Yunyun Zhao, Xuechun Fan, Lulu Tian, Xiangyan Li, Jia Mi

**Affiliations:** ^1^ College of Traditional Chinese Medicine, Changchun University of Chinese Medicine, Changchun, China; ^2^ Research Center of Traditional Chinese Medicine, The First Affiliated Hospital of Changchun University of Chinese Medicine, Changchun, China; ^3^ Department of Endocrinology, The First Affiliated Hospital of Changchun University of Chinese Medicine, Changchun, China; ^4^ School of Life Sciences, Zhejiang Chinese Medical University, Hangzhou, China; ^5^ Northeast Asia Research Institute of Traditional Chinese Medicine, Key Laboratory of Active Substances and Biological Mechanisms of Ginseng Efficacy, Changchun University of Chinese Medicine, Changchun, China

**Keywords:** ferroptosis, mechanism, sarcopenia, rhabdomyolysis, rhabdomyosarcoma, fatigue, myositis

## Abstract

Ferroptosis is characterized by the accumulation of iron and lipid peroxidation products, which regulates physiological and pathological processes in numerous organs and tissues. A growing body of research suggests that ferroptosis is a key causative factor in a variety of skeletal muscle diseases, including sarcopenia, rhabdomyolysis, rhabdomyosarcoma, and exhaustive exercise-induced fatigue. However, the relationship between ferroptosis and various skeletal muscle diseases has not been investigated systematically. This review’s objective is to provide a comprehensive summary of the mechanisms and signaling factors that regulate ferroptosis, including lipid peroxidation, iron/heme, amino acid metabolism, and autophagy. In addition, we tease out the role of ferroptosis in the progression of different skeletal muscle diseases and ferroptosis as a potential target for the treatment of multiple skeletal muscle diseases. This review can provide valuable reference for the research on the pathogenesis of skeletal muscle diseases, as well as for clinical prevention and treatment.

## 1 Introduction

As proposed by Dixon in 2012, ferroptosis is a novel method of regulatory cell death (RCD) (Dixon et al., 2012). Ferroptosis is characterized by the accumulation of iron ions and products of lipid peroxidation, which are different from the currently known mechanisms for cell death, such as apoptosis, pyroptosis, necrosis, and autophagy (Dixon et al., 2012; Latunde-Dada, 2017; Li J. et al., 2020). Ferroptosis, on the other hand, displays unique morphological changes, such as mitochondrial shrinkage, an increase in membrane density, and diminished or absent mitochondrial cristae (Dixon et al., 2012; Yu et al., 2017). As research advances, the interaction among amino acids, lipids, and iron metabolism is considered to be the key to ferroptosis, and a number of related signaling pathways, genes, proteins, and organelles have been identified (Chen et al., 2017; Badgley et al., 2020; Zhang H. et al., 2020; Venkatesh et al., 2020; Yu et al., 2020; Li Y. et al., 2021; Kong et al., 2021). Furthermore, genes associated with autophagy, such as *nuclear receptor coactivator 4* (*NCOA4*) and *fanconi anemia complementation group D2* (*FANCD2*), have also been discovered as key regulators of ferroptosis (Hou et al., 2016; Song et al., 2016). Meanwhile, numerous studies have demonstrated that ferroptosis plays a crucial role in cancer, diabetes mellitus, chronic kidney disease, heart failure, and other disease processes, offering a promising perspective for future clinical treatment (Chen X. et al., 2019; Li D. et al., 2020; Mao et al., 2021; Wang et al., 2022).

Skeletal muscle is the largest organ of the human body (accounting for about 40% of the body weight) (Janssen et al., 2000), which is crucial for maintaining body movements, posture and essential movements (such as swallowing and breathing), glucose intake and temperature regulation (Ferrannini et al., 1988; Meyer et al., 2002; Fluck and Hoppeler, 2003; Klaus et al., 2005; Shiozu et al., 2015; Kanezaki et al., 2021). Meanwhile, the storage of amino acids and the secretion of muscle cytokines are also the key functions of skeletal muscle (Wolfe, 2006; Argiles et al., 2016; Hoffmann and Weigert, 2017). The former can provide the substrate needed for the generation of energy and protein in the body (Garber et al., 1976; Perez-Sala et al., 1987; Perriello et al., 1997; Hyde et al., 2005), and the latter can participate in a versatile of physiological and pathological processes such as inflammation regulation, insulin sensitivity, tumor growth inhibition and cognitive improvement (Pedersen and Febbraio, 2008; Hong et al., 2009; Ellingsgaard et al., 2011; Hojman et al., 2011; Wrann et al., 2013). The importance of skeletal muscle bestows the life-threatening perniciousness on skeletal muscle diseases. Among the diseases, not only do sarcopenia, rhabdomyolysis (RML), rhabdomyosarcoma (RMS) and exhaustive exercise-induced fatigue (EEIF) and other skeletal muscle diseases seriously affect the quality of life and body function of patients, but they can also induce or develop into a variety of acute/chronic diseases, such as diabetes, fractures, hypercalcemia and acute kidney injury (AKI) (Kawasaki et al., 1998; Lima et al., 2008; Liu et al., 2014; Oliveira and Vaz, 2015; Tsekoura et al., 2017; Welch et al., 2020). Therefore, it is an urgent medical problem to clarify the pathogenesis of different skeletal muscle diseases and seek effective therapeutic targets. Recent studies have found that ferroptosis is an important RCD that induces skeletal muscle cell death and prevents skeletal muscle proliferation and differentiation (Guerrero-Hue et al., 2019; Ding et al., 2021; Huang et al., 2021). At the same time, ferroptosis is repeatedly reported to be associated with skeletal muscle disease processes such as sarcopenia, RML, RMS, and EEIF (Guerrero-Hue et al., 2019; Dachert et al., 2020; Huang et al., 2021; Xiao et al., 2022). The exact role of ferroptosis in these diseases, however, has not been systematically elucidated.

In this review, the biological mechanisms and key regulators of ferroptosis that have been published so far will be elaborated on. What will also be summarized is the research progress of ferroptosis in various skeletal muscle diseases and possible related therapeutic target strategies, aiming to provide valuable information and new directions for ferroptosis to participate in the pathogenesis and treatment of skeletal muscle diseases.

## 2 The regulatory mechanism of ferroptosis

### 2.1 Lipid peroxidation and ferroptosis

As shown in current research, ferroptosis is caused by the accumulation of lipid peroxides and their decomposition products (Dixon et al., 2012; Yang and Stockwell, 2016; Feng and Stockwell, 2018). It is therefore important to understand the mechanisms of lipid peroxide production and clearance for the purpose of comprehending ferroptosis regulation.

#### 2.1.1 Lipid peroxide production

Reactive oxygen species (ROS) are a group of highly active chemicals containing oxygen, including superoxide anion, hydrogen peroxide (H2O2), hydroxyl radical and peroxyl radical (Bayir, 2005; Yang et al., 2013). ROS produced in physiological process can regulate tissue homeostasis and transduce cell signal transduction (Rhee et al., 2000; Zhang H. et al., 2013; Zhang et al., 2016; Ferreira et al., 2018). The intracellular antioxidant system strictly monitors the generation and elimination of the ROS to maintain balance (He et al., 2017). When the oxidation and antioxidant systems are out of balance, a large amount of ROS produced will react with polyunsaturated fatty acids (PUFAs) in membrane phospholipids to form lipid peroxidation, which will change the fluidity and permeability of cell membrane and eventually lead to cell death (Farmer and Mueller, 2013; Catala and Diaz, 2016; Su et al., 2019). Ferroptosis, named for its iron ion dependence, is a typical representative of this cell death mode (Dixon et al., 2012; Yang and Stockwell, 2016). Fenton reaction, a non-enzymatic reaction mediated by iron, can promote the lipid peroxidation of PUFAs in membrane lipids by producing highly toxic hydroxyl radicals, thereby inducing ferroptosis (He et al., 2020). Enzymatic reaction is another important way to cause lipid peroxidation of PUFAs (Yamamoto, 1991; Lee et al., 2021). During enzymatic reaction, the esterification of free PUFAs and the insertion of these molecules into membrane phospholipids are achieved by Acyl-CoA synthetase long-chain family member 4 (ACSL4) and Lysophosphatidylcholine acyltransferase 3 (LPCAT3) (Dixon et al., 2015; Yuan et al., 2016a; Doll et al., 2017). Subsequently, lipoxygenase catalyzes lipid peroxidation of membrane phospholipids, which results in ferroptosis (Yang et al., 2016). Meanwhile, malondialdehyde (MDA) and 4-hydroxy-nonenal (4-HNE), which are produced during lipid peroxide degradation, can have adverse effects on the structure and function of proteins and nucleic acids (Ayala et al., 2014). Reducing lipid peroxidation has thus become a core step in the regulation of ferroptosis.

#### 2.1.2 Role of antioxidant system in ferroptosis

Lipid peroxidation mediated by ROS can lead to ferroptosis (Su et al., 2019). The three important intracellular antioxidant systems, GSH system, CoQ10 system and BH4 system, are essential for scavenging ROS and maintaining redox stability (Schafer and Buettner, 2001; James et al., 2004; Mugoni et al., 2013; Hatem et al., 2014; Jazvinscak Jembrek et al., 2014; Xue et al., 2017). In a variety of studies, they have also been proved to be a crucial defense to protect cells from lipid peroxidation induced ferroptosis (Bersuker et al., 2019; Kraft et al., 2020; Chen et al., 2022b). Hence, in the following discussion, we will focus on the role of these three antioxidant systems in ferroptosis.

##### 2.1.2.1 Glutathione system and ferroptosis

Glutathione (GSH) is a tripeptide composed of glutamate, cysteine and glycine, and also a paramount antioxidant in cells (Meister and Anderson, 1983). Glutathione peroxidase 4 (GPX4) is a selenoprotein with selenocysteine as its active center (Flohe et al., 1973; Rotruck et al., 1973; Ingold et al., 2018). GPX4 can convert toxic lipid hydroperoxides (L-OOH) into non-toxic lipid alcohol (L-OH) to prevent the accumulation of Fe^2+^ dependent lipid ROS on membrane lipids, thus playing a strong role in inhibiting ferroptosis (Imai and Nakagawa, 2003; Ursini and Maiorino, 2020). The transformation relies on the electrons provided by the process of GSH conversion into glutathione disulfide (GSSG) (Deponte, 2013). Subsequently, GSSG regenerates GSH under the catalysis of glutathione reductase (GR) and cofactor NADPH/H^+^, so as to continuously and circularly play an antioxidant role (Lu, 2009). Cysteine is the rate limiting substrate for GSH synthesis, and its availability directly affects the intracellular GSH level (Lu, 2009). Heavy chain solute carrier family 3 member 2 (SLC3A2, also known as CD98hc) and light chain solute carrier family 7 member 11 (SLC7A11, also known as XCT) construct the crucial amino acid transporter known as the cysteine/glutamate antiporter (system Xc^−^) (Bridges et al., 2012). Under system Xc^−^, cystine is exchanged 1:1 with intracellular glutamate and quickly reduced to cysteine for GSH synthesis and antioxidant defense (Mandal et al., 2010; Lewerenz et al., 2013). As a result, inhibiting the impairment of cystine uptake caused by system Xc^−^ can directly result in GSH depletion and GPX4 inactivation, which contributes to the induction of ferroptosis. Erastin is a representative of this ferroptosis trigger mechanism and has been widely used in ferroptosis induction experiments (Zhao Y. et al., 2020; Wang et al., 2020). Meanwhile, numerous researches results have proved that blocking System Xc^−^ to induce ferroptosis of cancer cells is an important mechanism for sorafenib to play an anti-cancer role (Li Y. et al., 2020; Li Z. J. et al., 2021), which provides a promising direction for inhibiting tumor growth.

In addition, in some mammalian cells, another way to maintain the supply of cysteine is the activation of transsulfuration pathway (McBean, 2012; Eriksson et al., 2015). Specifically, methionine, a sulfur donor from the diet, is converted into homocysteine (Mosharov et al., 2000; Sbodio et al., 2019). Then, homocysteine is condensed with serine under the catalysis of cystathionine β-synthase (CBS) to produce cystathionine, which finally produces cysteine through the action of cystathionine gamma-lyase (CSE) (Sbodio et al., 2019). It was found that cells with cysteinyl tRNA synthatase deletion can resist ferroptosis induced by erastin by up regulating genes and metabolites (cystathionine) in the transsulfuration pathway (Hayano et al., 2016). It is suggested that the transsulfuration pathway may be an alternative in inhibiting ferroptosis during cysteine deprivation. Further, cysteine produced by trans-sulfuration or uptake pathway combines with glutamate through GSH synthesis rate limiting enzyme glutamate cysteine ligase (GCL) (Lu, 2009). After the combination, GSH is finally synthesized under the catalysis of glutamate synthase (GSS) (Lu, 2009). The study found that GCL inhibition can induce ferroptosis of cancer cells (Nishizawa et al., 2018; Qin et al., 2021), contributing a new strategy for the development of cancer drugs.

##### 2.1.2.2 Coenzyme Q 10 system with ferroptosis

Coenzyme Q 10 (CoQ10, also known as ubiquinone) is a potent fat-soluble antioxidant that has been found in several recent studies to be involved in the regulation of ferroptosis (Laredj et al., 2014; Bersuker et al., 2019; Doll et al., 2019). Specifically, ferroptosis suppressor protein 1 (FSP1) is recruited to the plasma membrane by myristoylated and reduce CoQ10 to ubiquinol (CoQ10H2) by using NAD (P) H, thereby trapping lipid peroxidation free radicals to prevent lipid peroxidation (Bersuker et al., 2019). It is worth noting that although CoQ10 exists in almost all lipid membranes of cells, only CoQ10 outside mitochondria can inhibit ferroptosis under FSP1 dependent modification (Stockwell, 2019). Bersuker et al. found that FSP1 is a necessary factor to maintain tumor cell activity and growth under the condition of GPX4 knockout, and its expression is positively related to the resistance of cells to GPX4 inhibitors (Bersuker et al., 2019). It is indicated that FSP1/CoQ10/NAD(P)H pathway is a parallel and complementary regulatory mechanism of ferroptosis with GSH/GPX4 pathway.

The mevalonate pathway is essential for ferroptosis regulation because its metabolic intermediate, isopentenyl pyrophosphate, is indispensable for CoQ10 and GPX4 biosynthesis (Holstein and Hohl, 2004; Moosmann and Behl, 2004; Friedmann Angeli and Conrad, 2018). It was found that ferroptosis inducing 56 can induce ferroptosis by degrading GPX4 and consuming CoQ10, which is achieved by interfering with the mevalonate pathway (Shimada et al., 2016). Therefore, it is necessary to further explore the regulatory effect of mevalonate pathway in ferroptosis.

##### 2.1.2.3 Tetrahydrobioterin system and ferroptosis

Tetrahydrobioterin (BH4) has been found in recent studies to be a potent radical-trapping antioxidant that protects cells from ferroptosis by blocking lipid peroxidation transmission and acts independently of the GSH-dependent GPX4 protective pathway (Kraft et al., 2020; Soula et al., 2020). Guanosine triphosphate cyclohydrolase-1 (GCH1), the rate-limiting enzyme of BH4 synthesis, regenerates BH4 by catalyzing guanosine triphosphate (Thony et al., 2000). It was found that up-regulation of GCH1 restored the resistance of BH4-deficient cells to RSL3, indicating that GCH1 expression is decisive in the effectiveness of BH4 (Soula et al., 2020). In addition, BH4 may increase CoQ10 levels by converting phenylalanine into tyrosine, which provides a new perspective for its involvement in ferroptosis regulation (Kraft et al., 2020). However, the exact mechanism of BH4 system involved in the regulation of ferroptosis is still unclear, and further research is needed.

### 2.2 Iron metabolism and ferroptosis

Iron is a trace element necessary to maintain human life and health, and plays many essential physiological functions, including metabolism, oxygen transport, antioxidant reactions, electron transport, and DNA synthesis (Dlouhy and Outten, 2013). To maintain iron homeostasis, the body tightly regulates iron metabolism (including iron acquisition, utilization, storage, and efflux) (Wang and Pantopoulos, 2011). However, the destruction of iron homeostasis can lead to unstable iron accumulation and catalyze Fenton reaction, thus inducing ferroptosis (Winterbourn, 1995; Dixon et al., 2012; Henning et al., 2022). Therefore, regulation of iron metabolism is vital for the ferroptosis process.

#### 2.2.1 Cellular iron metabolism and ferroptosis

A complex formed by transferrin (TF) containing iron and the transferrin receptor (TFRC) on the cell membrane enters the endosome through endocytosis under physiological conditions (Andrews, 2000; Hentze et al., 2010). An endosome containing the complex is acidified to promote the release of Fe^3+^ from TF, which is then reduced to Fe^2+^ by six-transmembrane epithelial antigen of prostate 3 (Steap3) (Ohgami et al., 2005), and the iron is transported to the unstable labile iron pool in the cytoplasm by the divalent metal transporter 1 (DMT1) (Fleming et al., 1998). Studies have shown that TFRC and DMT1 overexpression can promote unstable iron accumulation and trigger lipid peroxidation, thus becoming key regulators of ferroptosis (Song et al., 2021a; Zhang H. et al., 2021; Guo et al., 2021; Lu et al., 2021). Additionally, the high expression of heat shock protein beta-1 (HSPB1) prevents ferroptosis by inhibiting TFRC-mediated iron absorption (Chen et al., 2006; Sun et al., 2015). Ferritin is the major cytoplasmic iron storage protein complex, which includes ferritin light chain (FTL) and ferritin heavy chain 1 (FTH1) (Knovich et al., 2009). Ferroptosis can be inhibited by the ferroxidase activity of FTH1, which converts Fe^2+^ captured by ferritin into Fe^3+^ in order to reduce the production of ROS (Theil, 2013). A number of studies have confirmed that high expression of FTH1 can reduce susceptibility to ferroptosis *in vivo* and *in vitro*, respectively (Tian et al., 2020; Kong et al., 2021). Mammalian cells release iron through ferroportin 1 (FPN1) (Nemeth et al., 2004; Ward and Kaplan, 2012). A high level of FPN1 expression can promote iron efflux and protect cells from ferroptosis (Zhao X. et al., 2020; Tian et al., 2021). The iron regulatory protein 1/2 (IRP1/2) can bind to the iron responsive element (IRE) to regulate the expression of various iron metabolism proteins (DMT1, TFRC, FPN, FTH1/FTL), which is responsible for regulating iron homeostasis in cells (Aziz and Munro, 1987; Koeller et al., 1989; Xu et al., 2018; Xu M. et al., 2022). By promoting the expression of IRP1/2, the ferroptosis inducers erastin and RSL3 increase the susceptibility of melanoma cells to ferroptosis (Yao et al., 2021).

In general, each regulatory link in cellular iron metabolism affects the intracellular iron content and therefore contributes to ferroptosis.

#### 2.2.2 Mitochondrial iron metabolism and ferroptosis

Iron metabolism in mitochondria is crucial to the control of cellular iron homeostasis. Iron transported from endosomes, cytosols, or ferritin traverses the outer membrane and inner membrane of mitochondria to reach the mitochondrial matrix, where it is utilized for the biosynthesis of heme and iron sulfur clusters or stored by mitochondrial ferritin (FtMt) to maintain cellular iron homeostasis (Paul et al., 2017). Mitoferrin 1/2 (Mfrn1/2) is a crucial component in facilitating iron transport across the inner mitochondrial membrane during this process (Paradkar et al., 2009). Studies have shown that reduced Mfrn expression may minimize ferroptosis by ameliorating mitochondrial iron excess (An et al., 2022; Zhang T. et al., 2022). Not only does FtMt store free iron in mitochondria, but it can also transport cytoplasmic iron to mitochondria, which is essential for controlling mitochondrial iron metabolism (Corsi et al., 2002; Drysdale et al., 2002). *In vivo* and *in vitro*, overexpression of FtMt abolished erastin-induced ferroptosis (Wang et al., 2016). In addition, the mitochondrial outer membrane protein CDGSH iron sulfur domain 1 (CISD1) was reported to reduce the sensitivity of liver cancer cells to erastin by inhibiting mitochondrial iron overload mediated lipid peroxidation (Yuan et al., 2016b), providing a new target for cancer treatment.

### 2.3 Heme metabolism and ferroptosis

**FIGURE 1:**
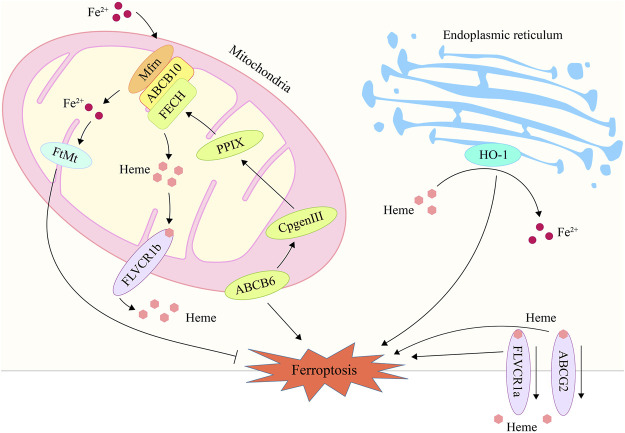
The processes of heme metabolism and mitochondrial iron metabolism are implicated in ferroptosis. In the endoplasmic reticulum, HO-1 degrades heme and releases iron ions, hence raising intracellular iron content and promoting ferroptosis. ABCB6 stimulates heme production by transferring CPgenIII from the cytosol to the mitochondrial membrane gap, a process that may play a role in ferroptosis. By exporting heme *via* the plasma membrane, ABCG2 and FLVCR1 contribute to the control of ferroptosis. Mfrn is capable of transporting iron through the inner membrane and into the mitochondrial matrix, hence elevating the iron concentration in mitochondria and inducing ferroptosis. FtMt may store free iron in mitochondria, hence reducing iron concentration and inhibiting ferroptosis. HO-1, heme oxygenase 1; ABCB6, ATP-binding cassette sub-family B member 6; ABCG2, ATP-binding cassette subfamily G member 2; Mfrn, mitoferrin; FtMt, mitochondrial ferritin.

Heme is the primary source of functional iron in the human body and a crucial component of erythropoiesis (Chung et al., 2012; Hooda et al., 2014). Therefore, the maintenance of systemic iron homeostasis requires the maintenance of a normal heme metabolism. The up-regulation of heme oxygenase 1 (HO-1), a crucial enzyme that degrades heme to liberate iron, can participate in ferroptosis induction by increasing intracellular iron levels (Tenhunen et al., 1968; Han et al., 2022). HO-1 inhibitor zinc protoporphyrin IX was discovered to counteract erastin-induced ferroptosis in HT-1080 fibrosarcoma cells (Kwon et al., 2015). HO-1-deficient proximal tubular cells, however, are very vulnerable to ferroptosis produced by erastin and RAS-selective lethal 3 (RSL3), which may be correlated to elevated heme levels after HO-1 deletion (Adedoyin et al., 2018). Heme metabolism related genes such as ATP-binding cassette subfamily B member 6 (ABCB6), Feline leukemia virus subgroup C receptor 1 (FLVCR1) and ATP-binding cassette subfamily G member 2 (ABCG2) were also found to be associated with ferroptosis (Tang et al., 2020; Zhang et al., 2020; Kawai et al., 2022). ABCB6 can transport the coproporphyrinogen III (CPgenIII) precursor of heme synthesis from the cytosol to the mitochondria, which is a key gene for heme synthesis (Krishnamurthy et al., 2006). As is shown in a research, low expression of ABCB6 in hepatocellular carcinoma may reduce the sensitivity of cancer cells to ferroptosis by reducing heme production (Zhang et al., 2020). Another study found that ABCB6 and FLVCR1 (an important carrier of cell surface heme output) were strongly positive and synergetic in tumor tissue, and were significantly down regulated after erastin intervention (Quigley et al., 2004; Keel et al., 2008; Tang et al., 2020). These results suggest that the heme content in cancer cells may affect their sensitivity to ferroptosis. In addition, as another important executor of heme export, part of ABCG2’s heme output capacity was found to be related to ferroptosis in recent studies (Desuzinges-Mandon et al., 2010; Kawai et al., 2022). But there is still no substantive research to support this finding yet. In general, heme metabolism is a promising research direction in the regulation mechanism of ferroptosis, which needs more attention and research (Figure 1; Table 1).

**TABLE 1:** Heme metabolism related genes and ferroptosis.

Gene	Protein name	Function	Relationship with ferroptosis	References
ABCB6	ATP binding cassette subfamily B member 6	Transport CPgenIII from the cytosol to the mitochondria to promote heme synthesis	Low expression of ABCB6 might promote ferroptosis through reducing iron consumption by inhibit heme synthesis	Krishnamurthy et al. (2006); Zhang et al. (2020); Tang et al. (2020)
FLVCR1(a/b)	Feline leukemia virus subgroup C receptor 1	Export heme	Low expression of FLVCR1 increases tumor cell sensitivity to erastin by reducing iron-containing heme export	Quigley et al. (2004); Keel et al. (2008); Tang et al. (2020)
ABCG2	ATP-binding cassette subfamily G member 2	Export heme	Regulation of heme output involved in ferroptosis	Desuzinges-Mandon et al. (2010); Kawai et al. (2022)
HO-1	Heme oxygenase 1	Breaks down heme to produce free iron, biliverdin and CO	Promotes free iron release to enhance cellular ferroptosis sensitivity	Tenhunen et al. (1968); Han et al. (2022); Kwon et al. (2015)
		Protect cells from oxidative stress	Ferroptosis is regulated by Nrf2/HO-1 axis	Niu et al. (2020); Chen et al. (2021b); Li et al. (2021b); Lv et al. (2021)

### 2.4 Amino acid metabolism and ferroptosis

Amino acid metabolism is closely related to ferroptosis, one of the main reasons is that it participates in GSH synthesis (Te Braake et al., 2008; Sikalidis et al., 2014; Sun et al., 2018; Xu Y. et al., 2022). In addition to the above-mentioned cysteine, glutamate is another essential amino acid for GSH synthesis, and glutaminolysis is one of its sources (Whillier et al., 2011). By encouraging the conversion of glutamine to glutamate and boosting the synthesis of GSH, glutaminase 2 (GLS2) improves the antioxidant capacity of cells (Xiang et al., 2013). The ferroptosis resistance of cardiomyocytes can be significantly increased by targeted regulation of GLS2, according to studies (Zhou et al., 2021). However, a new regulatory mechanism for glutamate neurotoxicity is provided by the fact that high glutamate levels cause ferroptosis in neuronal cells by interfering with cystine uptake (Olney, 1971; Murphy et al., 1989; Jiang et al., 2020). Additionally, it has been demonstrated that the downstream metabolite of glutaminolysis, α-ketoglutarate, is involved in the production of lipid ROS, raising the possibility that ferroptosis influenced by glutaminolysis (Gao et al., 2015). Meanwhile, compound 968, a glutaminolysis inhibitor, can significantly reduce erastin sensitivity under cystine deficiency (Gao et al., 2015), further demonstrated the necessity of glutaminolysis for ferroptosis. It is interesting to note that the ferroptosis inhibitor ferrostatin-1 (Fer-1) completely restored the sharply decreased glutamine level under RSL3 intervention (Rodriguez-Graciani et al., 2022). Based on the aforementioned findings, it is possible that the amino acid environment in which cells are located plays a role in the bidirectional regulation of ferroptosis by glutaminolysis.

Other amino acids, such as branched chain amino acids, tryptophan and lysine, is pivotal in ferroptosis (Chepikova et al., 2020; Wang K. et al., 2021; Zeitler et al., 2021). It was shown that the branched-chain amino acid aminotransferase two and lysine oxidase can participate in the ferroptosis process by antagonizing the system Xc^−^ inhibition and promoting H2O2 generation, respectively (Chepikova et al., 2020; Wang K. et al., 2021). Meanwhile, the tryptophan metabolite indole-3-pyruvate was recently found to negatively regulate ferroptosis through direct free radical depletion and upregulation of antioxidant genes (SLC7A11 and HO-1) (Zeitler et al., 2021). In addition, despite no relevant research report that arginine, serine and glycine participate in the regulation of ferroptosis, the three amino acids have shown the potential to fight ferroptosis in some studies (Possemato et al., 2011; Ye et al., 2014; Sen et al., 2018), which needs more exploration and excavation.

### 2.5 Other ferroptosis regulatory proteins

#### 2.5.1 Nuclear factor erythroid 2-related factor 2

Nuclear factor erythroid 2-related factor 2 (Nrf2) is a crucial transcription factor for cell antioxidants and a crucial ferroptosis regulator (Dodson et al., 2019; Anandhan et al., 2020). To stop lipid peroxides-mediated ferroptosis, Nrf2 can directly control the expression level of the GPX4 protein and important genes for GSH synthesis, such as the catalytic and regulatory subunits of glutamate cysteine ligase (GCLC/GCLM), GSS, GR, and XCT (Chan and Kwong, 2000; Madduma Hewage et al., 2017; Feng et al., 2021; Scibior et al., 2021; Lu et al., 2022). Meanwhile, with the ability of targeting key genes (*FTH1*, *FPN1*) that regulate iron metabolism, Nrf2 can participate in the process of ferroptosis by affecting intracellular iron levels (Harada et al., 2011; Liu et al., 2020). In addition, Nrf2 also regulate the key genes of heme metabolism *ABCB6*, *ABCG2*, *HO-1* and *heme responsive gene-1* to participate in the process of ferroptosis (Hubner et al., 2009; Singh et al., 2010; Campbell et al., 2013; Dong et al., 2020). Among them, HO-1 is not only the key enzyme to decompose heme, but also an important antioxidant (Tenhunen et al., 1968; Niu et al., 2020). Multiple studies have shown that activating the antioxidant response axis Nrf2/HO-1 is a crucial means of inhibiting ferroptosis and ameliorating myocardial ischemia-reperfusion injury, ulcerative colitis, and acute lung injury (Chen et al., 2021b; Li J. et al., 2021; Lv et al., 2021). Kelch-like ECH-associated protein 1 (Keap1) is a substrate adaptor protein of E3 ubiquitin ligase, which tightly controls the activity of Nrf2 by way of the ubiquitin-proteasome system (Furukawa and Xiong, 2005; Yamamoto et al., 2018). In order to prevent Nrf2 degradation and to promote its nuclear translocation and maintain cellular redox homeostasis, p62 can bind to Keap1 in a competitive manner (Tan et al., 2021). Studies have shown that one of the effective ways to treat liver cancer, endometrial hyperplasia, and protect neurons is by mediating the p62/Keap1/Nrf2 pathway to regulate ferroptosis (Sun et al., 2016; Zhang M. et al., 2021; Li et al., 2022). Owing to that Nrf2 is extensive in the regulation of ferroptosis, targeting Nrf2 is of great significance in the treatment of ferroptosis related diseases.

#### 2.5.2 P53

A crucial tumor suppressor gene called p53 is involved in the cell cycle, aging, apoptosis, and autophagy (Ong and Ramasamy, 2018). But more and more research has revealed that p53 also plays a significant part in controlling ferroptosis (Zhang Y. et al., 2021; Gao et al., 2021; Lei et al., 2021). Study has indicated that, as a direct target gene of p53, spermidine/spermine N1-acetyltransferase 1 (SAT1) can up-regulate arachidonate 15-lipoxygenase to promote ferroptosis due to accumulation of lipid peroxidation under ROS-induced stress (Ou et al., 2016). Other studies have shown that up-regulated p53 can affect GSH synthesis by inhibiting the expression of its downstream target gene SLC7A11, thereby inducing ferroptosis (Jiang et al., 2015; Guan et al., 2020). Meanwhile, by suppressing the expression of SLC7A11, p53 can indirectly activate the positive regulator of ferroptosis arachidonate 12-lipoxygenase (ALOX12) to promote ferroptosis (Chu et al., 2019). Additionally, p53 can facilitate dipeptidyl-peptidase 4’s (DPP4) nuclear translocation and join forces with it to form the DPP4-p53 complex, which lowers DPP4-dependent lipid peroxidation and inhibits ferroptosis in colorectal cancer cells (Xie et al., 2017). It has been suggested that p53 may mediate glutaminolysis to take part in the ferroptosis process because the essential enzyme for glutamine catabolism, GLS2, is also a direct target gene of p53 (Hu et al., 2010; Suzuki et al., 2010).

#### 2.5.3 Nuclear receptor coactivator 4

One important mechanism for ferritin degradation is ferritinophagy, which can encourage the release of iron and provide a substrate for ferroptosis (Gao et al., 2016; Hou et al., 2016). A crucial regulator of ferritinophagy, NCOA4, can specifically bind to the surface arginine of FTH1 and promote ferritin degradation by lysosomes and autophagosomes (Mancias et al., 2014; Gryzik et al., 2017). Ferritinophagy also involves the traditional *autophagy-related gene 3* (*Atg3*), *Atg5*, and *Atg7*, which are significant players (Hou et al., 2016). Knockout of Atg5, Atg7 or NCOA4 can effectively inhibited ferritin degradation to reduce erastin-induced ferroptosis (Hou et al., 2016). Furthermore, a new ferroptosis inhibitor compound 9a was found to block ferroptosis by disrupting the interaction of NCOA4-FTH1 (Fang et al., 2021), which opens up a new access for the development of ferroptosis inhibitors.

#### 2.5.4 Fanconi anemia complementation group D2

As a nuclear protein involved in DNA damage repair, FANCD2 was discovered to be a key gene in autophagy-dependent ferroptosis (Nakanishi et al., 2002; Miao et al., 2022). Furthermore, FANCD2 mediates ferroptosis independently of autophagy (Song et al., 2016). The deletion of FANCD2 increases iron overload and lipid peroxidation in erastin-induced ferroptosis of bone marrow mesenchymal stem cells, which is associated with restricted expression of GPX4, FTH1, and upregulation of TFR1 (Song et al., 2016). It offers a novel approach to alleviating the side effects of bone marrow damage brought on by cancer treatment.

#### 2.5.5 CDGSH iron sulfur domain 1

**FIGURE 2:**
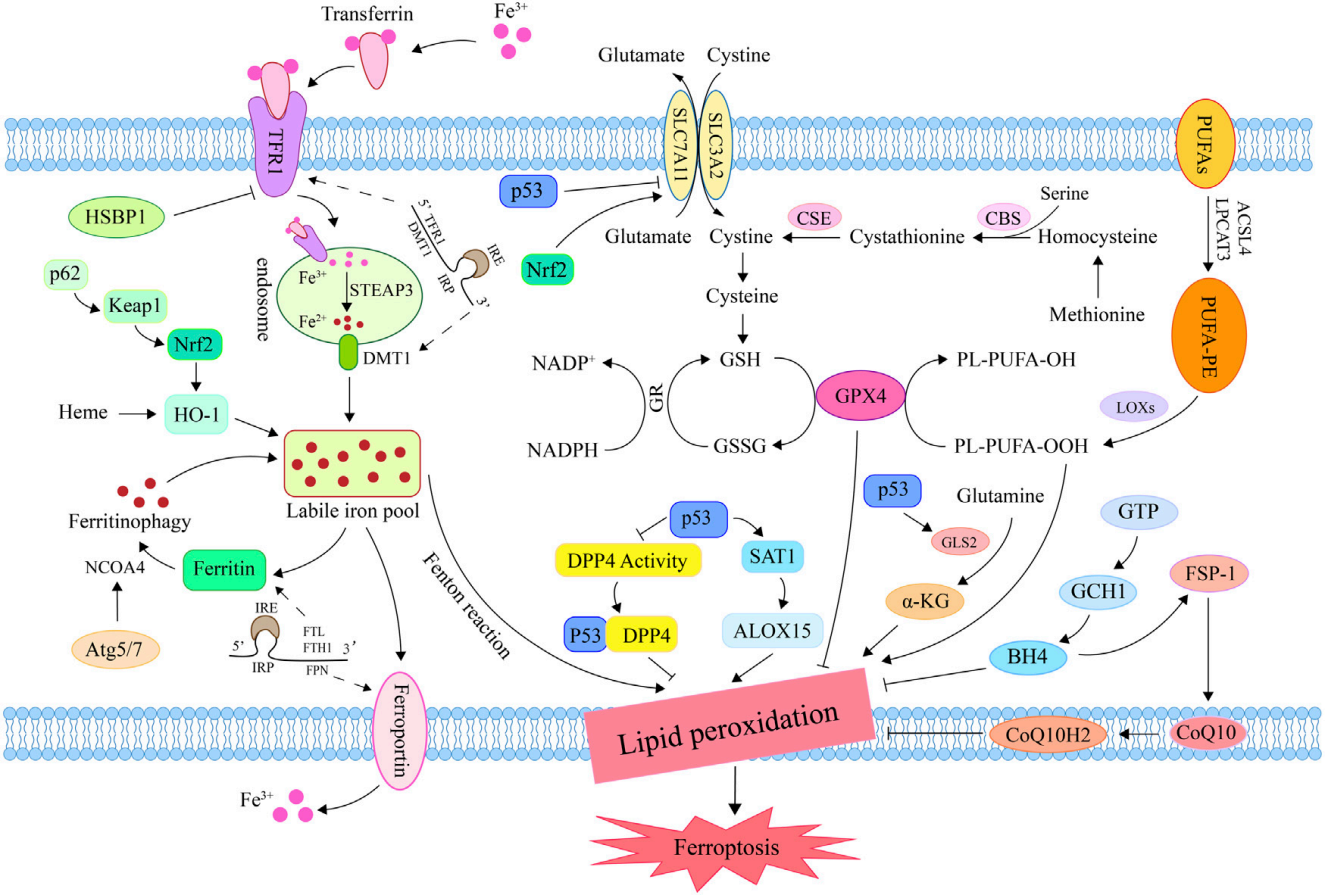
Mechanisms for regulating ferroptosis. Iron in circulation binds to TFR1 and enters the endosome *via* endocytosis; then, steap3 converts Fe^3+^ to Fe^2+^. DMT1 transports Fe^2+^ into the lip in order to promote unstable iron accumulation and induce ferroptosis *via* the Fenton reaction. Methionine generates cysteine under the action of CBS and CSE for GSH synthesis. Inhibition of system Xc^−^ reduced GSH synthesis and inactivated GPX4, promoting lipid peroxide accumulation and inducing ferroptosis. Under the catalysis of ACSL4, LPCAT3, and LOX, PUFA lipid peroxidation resulted in ferroptosis. By promoting iron release, the ferritin autophagy-related genes Atg5, Atg7, and NCOA4 induce ferroptosis. By influencing genes related to iron/heme metabolism and amino acid metabolism, Nrf2 plays a significant role in the regulation of ferroptosis. By inhibiting DPP4 activity and systemic SLC7A11 expression, or by activating SAT1 and GLS2, P53 can induce ferroptosis. FSP1 prevents lipid peroxidation by converting CoQ10 to CoQ10H2, thereby inhibiting ferroptosis. GCH1 blocks the chain propagation of lipid peroxidation by catalyzing GTP to generate BH4. TFR1, transferrin receptor 1; DMT1, divalent metal transporter 1; CBS, cystathionine β-synthase; CSE, cystathionine gamma-lyase; GSH, glutathione; GPX4, glutathione peroxidase 4; ACSL4, Acyl-CoA synthetase long-chain family member 4; LPCAT3, lysophosphatidylcholine acyltransferase 3; LOX, lipoxygenase; PUFA, polyunsaturated fatty acid; Atg5, autophagy-related gene 3; Atg7, autophagy-related gene 7; NCOA4, nuclear receptor coactivator 4; Nrf2, nuclear factor erythroid 2-related factor 2; DPP4, dipeptidyl-peptidase 4; SLC7A11, solute carrier family 7 member 11; SAT1, spermidine/spermine N1-acetyltransferase 1; GLS2, glutaminase 2; FSP1, ferroptosis suppressor protein 1; CoQ10, ubiquinone; CoQ10H2, ubiquinol; GCH1, GTP cyclohydrolase-1; GTP, Guanosine triphosphate; BH4, tetrahydrobioterin.

Recent research indicates that the outer mitochondrial membrane protein CDGSH iron sulfur domain 1 (CISD1) can inhibits mitochondrial iron uptake and lipid peroxidation, thereby negatively regulating erastin-induced ferroptosis (Yuan et al., 2016b). Meanwhile, pioglitazone inhibits iron-mediated mitochondrial lipid peroxidation and subsequent ferroptosis by binding CISD1 and stabilizing iron-sulfur clusters (Yuan et al., 2016b). Another study showed that the ferroptosis-related gene CISD1 is anticipated to become one of the novel biomarkers for predicting the prognosis of breast cancer patients, thereby providing a new target for cancer therapy (Wang D. et al., 2021) (Figure 2).

## 3 The role of ferroptosis in skeletal muscle diseases

Ferroptosis has been widely concerned by researchers since its discovery, and has proved to play a pivotal role in the progress of human diseases in various systems, such as tumors, cardiovascular and cerebrovascular diseases, nervous system diseases, respiratory diseases and digestive diseases (Do Van et al., 2016; Fang et al., 2019; Badgley et al., 2020; Ma et al., 2020b; Guan et al., 2020; Bao et al., 2021; Wu et al., 2021; Zheng et al., 2021; Liu T. et al., 2022; Bao et al., 2022; Zhang Y. et al., 2022). In recent years, the functions of ferroptosis in a variety of skeletal muscle diseases has attracted the attention of researchers, and has been reported to be an overriding participant in the physiological and pathological processes such as sarcopenia, RML, RMS, and EEIF (Guerrero-Hue et al., 2019; Dachert et al., 2020; Huang et al., 2021; Xiao et al., 2022). Therefore, we summarized the functions of ferroptosis in the pathogenesis of these skeletal muscle diseases.

### 3.1 Ferroptosis and sarcopenia

Sarcopenia is an age-related degenerative loss of skeletal muscle strength and quality (Cruz-Jentoft et al., 2010). The main mechanism of its occurrence and development is the imbalance of muscle synthesis and degradation (Tan et al., 2020a), which is closely related to the decline of satellite cells (SCs) number/function (Brack et al., 2005; Budai et al., 2018). SCs are embryonic muscle stem cells located beneath the basal layer of muscle fibers and are in a quiescent state (Mauro, 1961; Yin et al., 2013). When muscle is damaged, SCs are activated and will proliferate into myoblasts, which will then differentiate and fuse to exert a powerful ability to promote muscle regeneration and repair damage (Aziz et al., 2012; Chen F. et al., 2019). However, some researches indicates that the number and function of SCs decline significantly with age, which would severely impair their capacity for self-renewal and regeneration and would lead to sarcopenia (Day et al., 2010; Jang et al., 2011; Sousa-Victor et al., 2014). Previous research has demonstrated that unstable iron accumulation existed in aging skeletal muscle and could promote muscle damage in mice by down-regulating SCs markers (paired box 7, myogenic differentiation antigen and myogenic factor 5) and inhibiting C2C12 myoblast differentiation (Ikeda et al., 2019). Another study found changes in ferroptosis related factors such as HO-1, SAT1 and prostaglandin-endoperoxide synthase 2 (Ptgs2) in muscle samples of elderly people with sarcopenia (Ding et al., 2021). It is suggested that SCs and C2C12 myoblasts may participate in sarcopenia disease through ferroptosis, which has been verified in several recent studies.

**FIGURE 3:**
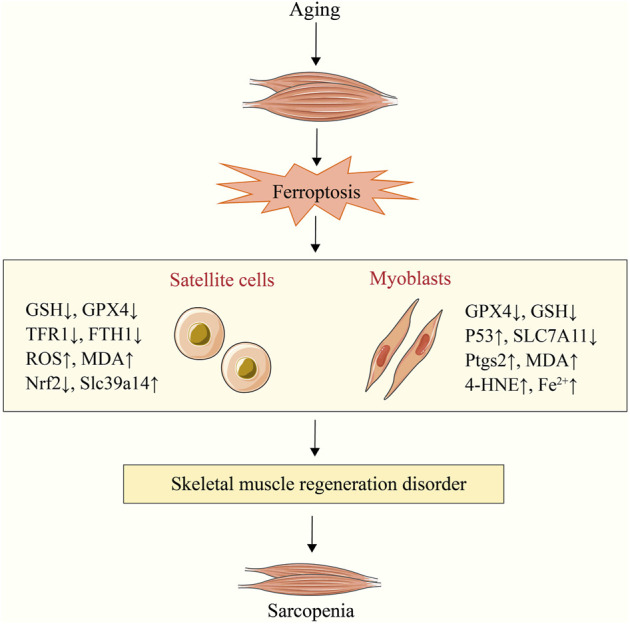
Sarcopenia caused by satellite cell and myoblast ferroptosis. Aging can result in satellite cell and myoblast ferroptosis. Sarcopenia is caused by the subsequent dysfunction of skeletal muscle regeneration. NADPH, nicotinamide adenine dinucleotide phosphate; Ptgs2, prostaglandin-endoperoxide synthase 2; 4-HNE, 4-hydroxynonenal; Slc39a14, zinc transporter Zip14; MDA, malondialdehyde; SLC7A11, solute carrier family 7 member 11; TFR1, transferrin receptor 1; GPX4, glutathione peroxidase 4; GSH, glutathione.

TFR1 is an important factor in the activation, proliferation, and maintenance of SCs, whereas the expression of TFR1 is significantly decreased in aging skeletal muscle (DeRuisseau et al., 2013; Ding et al., 2021). The research demonstrates that deletion of the *TFR1* gene can promote zinc transporter Zip14 (Slc39a14) to absorb non-heme iron and down-regulate GPX4, Nrf2, and FTH1 to increase unstable iron accumulation and lipid peroxidation level, thereby inducing ferroptosis in skeletal muscle cells and impairing their regeneration (Ding et al., 2021). As is shown in another study, in animal models with sarcopenia, C2C12 myoblasts had age-related iron accumulation (Huang et al., 2021). Simultaneously, the activation of p53/SLC7A11 axis can induce ferroptosis of C2C12 myoblasts to hinder their differentiation into myotubes and to promote the progression of sarcopenia, which can be reversed by the ferroptosis inhibitors fer-1 and DFO (Huang et al., 2021). Targeted regulation of SCs/myoblast ferroptosis is therefore anticipated to become a new treatment strategy for sarcopenia. In addition, the CSE derivative hydrogen sulfide can inhibit the acetylation modification of ALOX12, prevent lipid peroxidation of phospholipid membrane, and protect myoblasts from ferroptosis (Wang Y. et al., 2021). It provides a new target for preventing ferroptosis in aging skeletal muscle, although the relationship to primary sarcopenia was not explored (Figure 3).

### 3.2 Ferroptosis and rhabdomyolysis

RML is an acute clinical syndrome caused by the injury of skeletal muscle cells and the release of intracellular components (such as myoglobin and creatine kinase) into the systemic circulation (Lindner and Zierz, 2003; Stahl et al., 2020). It is exhibited in study that ferroptosis of skeletal muscle cells induced by the inhibition of the antioxidant axis Nrf2-XCT/GPX4 is one of the potential mechanisms of atorvastatin-induced muscle-related symptoms (muscle weakness, pain, cramps, and RML) (Zhang Q. et al., 2022). It suggests that ferroptosis may be related to the disease progression of RML. AKI is a common complication of RML and one of the leading causes of death in RML patients (Ahmad et al., 2021). A study reported that iron deficiency could exacerbate RML-induced AKI by evoking lipid peroxidation through catalytic heme-iron (Zhao et al., 2021b). Another study found that the related characteristics of ferroptosis include decreased GSH levels and the accumulation of lipid peroxidation products MDA, 4-HNE and iron occurred in RML mice (Guerrero-Hue et al., 2019). Simultaneously, fer-1 has a significant improvement effect on muscle cell death, renal function and structure of RML mice compared to zVAD (an apoptosis inhibitor) and RIPK3-knockout mice (necroptosis pathway deficiency) (Guerrero-Hue et al., 2019). These results suggest that ferroptosis is a paramount pathogenic factor in RML and its associated renal injury. Furthermore, curcumin, a powerful antioxidant, has been reported to improve the AKI associated with RML by increasing the ferroptosis resistance of cells (Guerrero-Hue et al., 2019), which opens a new way for the treatment of RML syndrome.

RML is also one of the major types of exertional heat stroke (EHS)-related muscle damage (Epstein and Yanovich, 2019; Laitano et al., 2021). The experimental results revealed that high levels of iron content, ferroptosis markers (Ptgs2, MDA), and typical ferroptotic mitochondrial morphological changes would happen in the muscle tissue of RML mice after EHS (He et al., 2022). Through further study *in vivo* and *in vitro*, it is found that post-EHS-mediated ferroptosis of skeletal muscle cells depended on the up-regulation of ACSL4, the key gene for lipid modeling, and rosiglitazone (ACSL4 inhibitor) treatment could significantly reduce the skeletal muscle injury caused by EHS (He et al., 2022). Inhibition of ferroptosis by targeting ACSL4, therefore, may be a novel approach to prevent RML after EHS.

### 3.3 Ferroptosis and rhabdomyosarcoma

**FIGURE 4:**
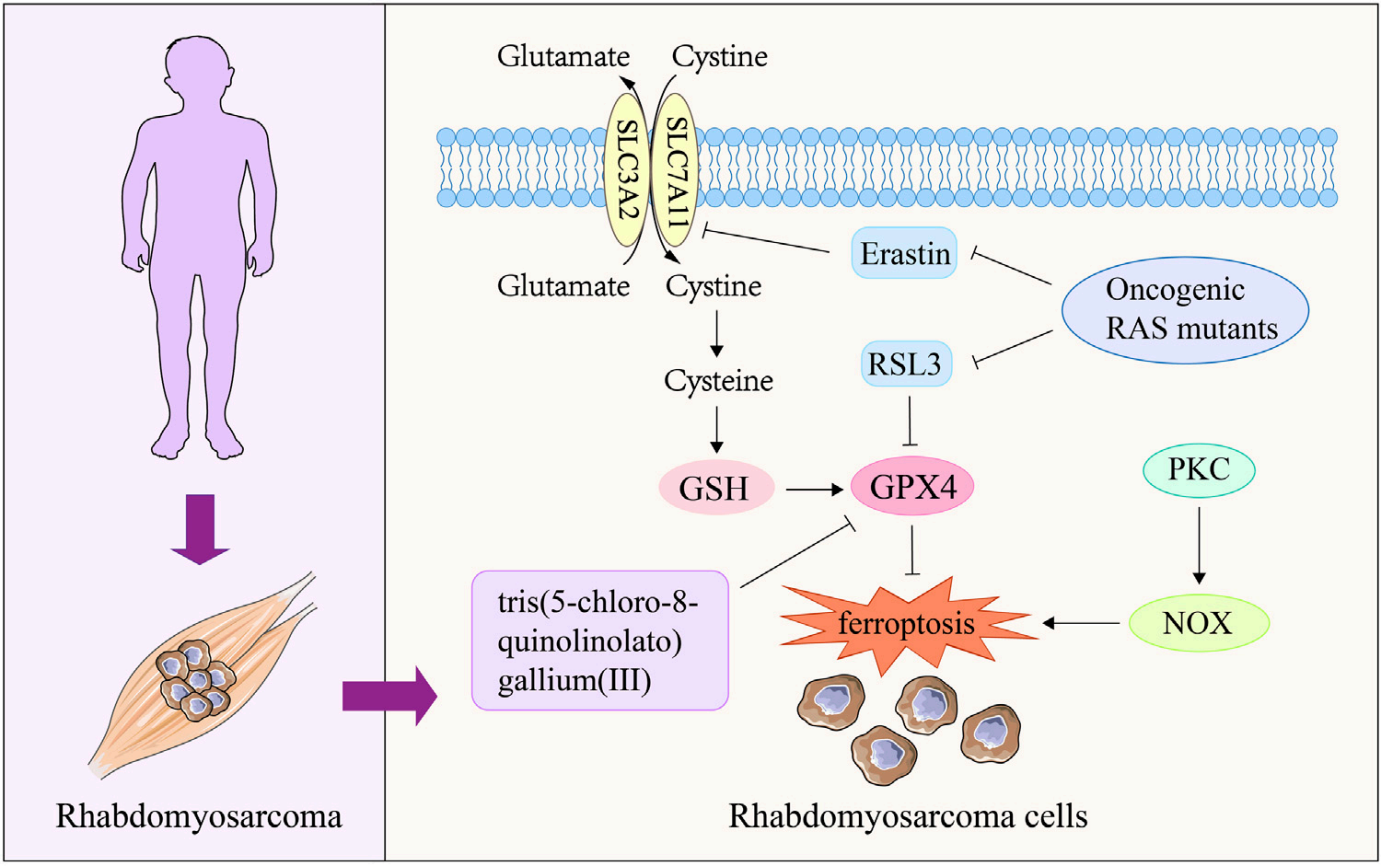
The role of ferroptosis in rhabdomyosarcoma. Oncogenic RAS mutants are the key mediator of RMS disease, which can block the ferroptosis of RMS cells induced by erastin and RSL3. The complex tris(5-chloro-8-quinolinolato) gallium (III) can induce ferroptosis in RMS cells by reducing GPX4 expression, thereby exerting therapeutic effects on RMS. The activation of PKC-NOX pathway can participate in the process of RMS disease by increasing the ferroptosis resistance of RMS cells. PKC, protein kinase C; NOX, NADPH oxidases; GPX4, glutathione peroxidase 4; GSH, glutathione.

Characterized by poor survival and high recurrence, RMS is a malignant soft tissue tumor that occurs mostly in children and adolescents (Kramer et al., 1983; Ognjanovic et al., 2009; Dantonello et al., 2013; Egas-Bejar and Huh, 2014). Recent study has reported that RMS is sensitive to oxidative stress (Chen et al., 2013). Ferroptosis, as a novel mode of cell death induced by oxidative stress (Wu et al., 2018), has received attention in current RMS research. Research shows that erastin and RSL3, as common ferroptosis-inducing compounds, can cause ferroptosis of RMS cells through GSH consumption and GPX4 inactivation respectively (Codenotti et al., 2018; Dachert et al., 2020). Notably, inhibition of protein kinase C (PKC) isoform PKCα and its downstream target gene NADPH oxidases (NOX) isoforms (NOX1/4) can significantly protect RMS cells from erastin-induced ferroptosis, providing a new perspective for the treatment of RMS (Dachert et al., 2020). Activation of RAS-related signaling pathways is a key cause of RMS occurrence and recurrence (Zhang et al., 2013; Shern et al., 2014). It was found that ectopic expression of oncogenic RAS mutants (NRAS12V, KRAS12V and HRAS12V) significantly reduced the sensitivity of RMS13 cell line to erastin and RSL3 (Schott et al., 2015). This suggests that one of the pathways by which RAS drives the occurrence and progression of RMS is to confer ferroptosis resistance to RMS cells. In addition, a recent work found that tris (5-chloro-8-quinolinololato) gallium (III) complex had an active anti proliferation effect on RMS cells, and its efficacy decreased under the co incubation of fer-1 (Hreusova et al., 2022). Meanwhile, cells treated with tris (5-chloro-8-quinolinololato) gallium (III) complex presented typical characteristics of ferroptosis, such as down-regulation of GPX4 expression and accumulation of lipid peroxide (Hreusova et al., 2022). These results suggest that ferroptosis may be a potential mechanism for this compound to exert RMS therapeutic effect. In general, targeting RMS cell ferroptosis is a promising researching direction in clinical treatment. However, at present, the research on ferroptosis and RMS is mainly conducted *in vitro*, and further intervention on ferroptosis *in vivo* is needed to fully investigate the relationship between ferroptosis and RMS (Figure 4).

### 3.4 Ferroptosis and exhaustive exercise-induced fatigue

EEIF refers to the inability of muscles to generate force due to prolonged and/or strenuous exercise, which not only reduces quality of life, but also promotes the development of fatigue itself, and even causes organic changes (Dalsgaard et al., 2004; da Rocha et al., 2018; Hou et al., 2020). Under physiological conditions, skeletal muscle fibers continuously produce ROS at a slow rate and increase during muscle contraction, which will be offset by the antioxidant system to maintain the balance of its production and removal (Reid, 2008). However, it is found in many research that long and/or strenuous exercise will cause a sharp ROS increase in skeletal muscle (Davies et al., 1982; Reid et al., 1992). Meanwhile, this activity dependent increase of ROS will induce lipid peroxidation of cell membrane and damage the healthy tissue, thus aggravating muscle fatigue during strenuous exercise (Dillard et al., 1978; Alessio et al., 1988). Therefore, ferroptosis may be a new type of cell death that participates in the physiological and pathological process of EEIF except apoptosis (Sun Y. et al., 2016; Liu S. et al., 2022). Excitingly, a recent report explored the relationship between ferroptosis and EEIF (Xiao et al., 2022). The experimental data showed that there were characteristics related to ferroptosis in skeletal muscle of EEIF mice, such as accumulation of iron, lipid peroxide, decreased expression of GPX4 and GSH, suggesting that ferroptosis may participate in the process of EEIF disease (Xiao et al., 2022). Trilobatin, as a natural food additive, has been reported to be anti-fatigue role by reducing the production of ROS and MDA, increasing the activity of GPX4 and the level of GSH, which is related to the inhibition of ferroptosis by the activation of Nrf2/ARE signaling pathway (Xiao et al., 2022). Based on the above, the high-level ROS produced in skeletal muscle after prolonged and/or strenuous exercise may increase the loss of muscle cells by activating ferroptosis, thereby promoting EEIF. Although there is limited evidence to support this view, it provides a new direction for the treatment of EEIF, which is worthy of more in-depth exploration from researchers in related fields.

### 3.5 Ferroptosis and idiopathic inflammatory myopathies

IIMs are a group of autoimmune diseases characterized by muscle inflammation (Parkes et al., 2015; Lundberg et al., 2016). Polymyositis (PM) and dermatomyositis (DM) are the two most common types of IIMs with high mortality (Yang et al., 2020). However, the role of ferroptosis in IIMs remains unclear. Vitamin E and selenium are important antioxidants that prevent lipid peroxidation with the effect of resisting ferroptosis (Conrad and Proneth, 2020; Hu et al., 2021; Tuo et al., 2021). It was found that a patient with chronic malabsorption and selective IgA deficiency lacking vitamin E and selenium appeared PM when receiving iron glucan treatment, which was related to lipid peroxidation caused by free iron activated free radicals (Foulkes et al., 1991). Ferritin is the main site of iron storage in the body, and elevated levels indicate iron accumulation in the body (Cook et al., 1974; Harrison, 1977; Worwood, 1987). Many population surveys based on PM/DM have found that the severity and prognosis of PM/DM and its complications (interstitial lung disease) were related to ferritinemia (Gono et al., 2010; Kawasumi et al., 2014; Ishizuka et al., 2016). In addition, mitochondrial dysfunction, as a landmark event of termination of ferroptosis and the main cause of ROS accumulation, has been reported as an important pathogenic mechanism of IIMs (Meyer et al., 2017; Boehler et al., 2019). Based on this, it is logical to speculate that ferroptosis is involved in the occurrence and development of IIM, and further exploration is needed to clarify the exact role ferroptosis plays in IIM (Table 2).

**TABLE 2:** Therapeutic strategies for skeletal muscle diseases associated with ferroptosis.

Target	Protein/reagent	Mechanisms	Diseases	References
Inducers				
GPX4	RSL3, tris(5-chloro-8-quinolinolato) gallium (III)	Inhibits GPX4, leading to accumulation of lipid peroxides	RMS	Codenotti et al. (2018); Hreusova et al. (2022)
SLC7A11	P53, erastin	Decreased cystine uptake, causing GSH depletion	Sarcopenia, RMS	Huang et al. (2021); Codenotti et al. (2018); Dachert et al. (2020)
Nrf2	Atorvastatin	Inhibits Nrf2, increases lipid peroxidation levels	RML	Zhang et al. (2022e)
Inhibitors				
Nrf2	Trilobatin	Promotes Nrf2 nuclear translocation, increases GSH levels	EEIF	Xiao et al. (2022)
ACSL4	Rosiglitazone	Down-regulate ACSL4 and reduce the production of lipid peroxidation	RML	He et al. (2022)
Iron	DFO	Chelates iron	Sarcopenia	Huang et al. (2021)
Lipid peroxidation	Fer-1	Inhibition of lipid peroxidation	Sarcopenia, RML, RMS	Huang et al. (2021); Guerrero-Hue et al. (2019); Hreusova et al. (2022)
PKCα	Gö6976	Inhibits the expression of PKCα	RMS	Dachert et al. (2020)
NOX1/4	GKT137831	Inhibits NOX1/4, reduce the production of lipid peroxidation	RMS	Dachert et al. (2020)

## 4 Conclusion and prospect

This review summarizes the regulatory mechanism of ferroptosis and its role in the progression of different skeletal muscle diseases. As mentioned above, in addition to the three classical regulatory pathways for ferroptosis in lipid, iron, and amino acid metabolism, a number of signal regulators and autophagy-related genes, such as *Nrf2, p53*, *NCOA4*, *FANCD2*, and *CISD1*, are also essential ferroptosis players. With the deepening of research, ferroptosis has been proved to be overriding in some muscle mass and dysfunction diseases, including sarcopenia, RML and EEIF. Skeletal muscle, composed of skeletal muscle cells, is an important organ to maintain human posture, exercise, energy metabolism and secretion of muscle cytokines. Therefore, it is of great significance to inhibit the ferroptosis of skeletal muscle cells in the treatment of sarcopenia, RML, EEIF and other diseases. However, in terms of RMS and other malignant tumor tissues, the key to prevent cancer occurrence and recurrence is to actively promote the ferroptosis of cancer cells. It is concluded that unstable iron accumulation, increase of lipid peroxide, inactivation of GPX4, inhibition of system Xc^−^ and depletion of GSH are common causes of ferroptosis in skeletal muscle diseases. The regulatory mechanisms and targets involved include P53/SLC7A11 axis, Nrf2-xCT/GPX4 axis, TFR1, ACSL4, PKCα and NOX1/4. These ferroptosis related targets were found to be an important way in distinct skeletal muscle disease treatment drugs to inhibit ferroptosis (Trilobatin and Rosiglitazone) or induce ferroptosis [tris (5-chloro-8-quinolinolato) gallium (III) and atorvastatin]. Their effects were similar to those of known ferroptosis interventions such as erastin, RSL3, and fer-1. However, it is not clear whether these regulatory factors are specific targets of therapeutic drugs for skeletal muscle diseases.

Due to the limitation of current literature, our review of ferroptosis and skeletal muscle disease is not very comprehensive. However, there are still some exploratory suggestions worth putting forward. Ferroptosis is a regulatory mechanism that is promising for the treatment of sarcopenia, RML, RMS and EEIF. Whereas the exploration of the correlation between these diseases and ferroptosis is still in its infancy. Meanwhile, ferroptosis may also be a breakthrough for us to overcome the problems in treatment skeletal muscle-related intractable diseases such as IIMs. Of note, in the treatment of RMS and other skeletal muscle diseases, it is necessary to properly promote and inhibit ferroptosis to intervene the disease process. Therefore, when treating cancer and skeletal muscle related diseases with ferroptosis as a target, we should pay attention to balancing the two-way effects of ferroptosis treatment drugs on cancer tissues and healthy tissues. This is especially critical for the treatment of cancer patients with skeletal muscle disease, and it is also the focus and difficulty in drug development. Moreover, several genes associated with heme metabolism, such as ABCB6, FLVCR1, and ABCG2, appear to significantly influence ferroptosis, providing profound reference for the study on ferroptosis regulatory targets in skeletal muscle diseases, which needs more exploration and excavation.
